# Supplementary material for: Comparison of Galleria mellonella, epithelial cell cytotoxicity, and mouse model of bacteremia to measure Pseudomonas aeruginosa virulence
Source: Infect Immun. 2026 Aug 19;94(9):e00335-26. doi: 10.1128/iai.00335-26 (PMC13552133; doi:10.1128/iai.00335-26)
Supplement: Supplementary file 1 [file iai.00335-26-s0002.docx]

**Supplemental Figure 1. Examples of the methodology for quantifying *P. aeruginosa* virulence by time-to-death in *G. mellonella*.** (A) Measurements of time to death of *G. mellonella* following bacterial injection. Three representative *P. aeruginosa* isolates were each injected at a dose of ~2,500 CFU into ten *G. mellonella* larvae. The time of death of each larva was recorded to calculate time to 50% mortality (LT_50_). (B) Generation of LT_50_ vs. ln(CFU) curves. Injections were repeated with additional doses of bacteria, and the LT_50_ values were plotted against the natural log of their respective doses for each isolate. A linear regression line was fit to the data points to allow for the calculation of an extrapolated LT_50_ value for a desired bacterial dose.

**Supplemental Figure 2. Concordance between virulence measurements in mice and *G. mellonella* at 300 CFU and 30,000 CFU.** The extrapolated LT_50_ values at a dose of (A) 300 CFU and (B) 30,000 CFU in *G. mellonella* vs. corresponding LD_50_ values from the murine bacteremia model. For each dose, a moderate to strong correlation between the two models was observed: Spearman’s rank correlation coefficient (ρ) = 0.75 (*P* < 0.0001) for 300 CFU, and ρ = 0.64 (*P* < 0.0001) for 30,000 CFU. Each symbol represents an individual isolate.

**Supplemental Figure 3. The ten most common *P. aeruginosa* sequence types (STs) in the NCBI genome repository.** Bars show the relative abundance of NCBI genomes available for each of the 10 most commonly deposited STs (NCBI was accessed in May 2023.) For each ST, it is indicated whether HRC status is supported by the literature and whether it is represented in our collection of 105 isolates.

**Supplemental Figure 4. Phylogenetic tree showing seven genetically related clusters of *P. aeruginosa* isolates.** A core genome phylogenetic tree was generated for the isolates included in the cluster analysis. Each color represents one of the seven defined phylogenetic clusters. Gray circles (cluster designated -1) indicate singleton isolates that did not cluster with any other groups. PABL043 (a PA7-like isolate) was excluded from this tree due to its substantial genetic divergence from the other isolates.

**Supplemental Figure 5. Phylogenetic distribution of virulence across infection models.** A maximum-likelihood core genome phylogenetic tree is shown, with concentric rings indicating (A) mouse LD_50_ values and (B) *G. mellonella* LT_50_ values at 3,000 CFU. *In vitro* secretion of T3SS effectors ExoS and ExoU is indicated by tips with filled circles (secretion-positive) or open circles (secretion-negative). PABL043 (a PA7-like strain) is excluded from this tree because it is genetically highly divergent from the other isolates.

**Supplemental Figure 6. *P. aeruginosa* isolates with the greatest discordance in relative virulence rank in the mouse and *G. mellonella* models.** Each point represents a single isolate plotted according to its relative virulence rank in mice and *G. mellonella*. Triangle orientation indicates isolates that exhibited greater virulence in G. mellonella (up) or mice (down).

**Supplemental Figure 7. Phylogenetic distribution of *P. aeruginosa* isolates with the greatest virulence rank difference between the mouse and *G. mellonella* models.** A maximum-likelihood core genome phylogenetic tree was generated. Concentric rings annotate T3SS effector secretion phenotypes and those isolates that exhibit the greatest virulence rank difference in the mouse and *G. mellonella* models.

**Supplemental Figure 8. Comparison of cell co-culture cytotoxicity and mouse virulence among ExoU^+^ isolates.** A549 cell lysis values are plotted against corresponding LD_50_ values from the murine bacteremia model for ExoU^+^ isolates, yielding a Spearman’s rank correlation coefficient (ρ) of -0.31 (*P* < 0.0001). Each isolate secreted only ExoU (orange circles) except for a single isolate that secreted both ExoU and ExoS (black square).

**Supplemental Table 1. *P. aeruginosa* isolates with the greatest rank difference in virulence between the mouse and *G. mellonella* models.** Virulence ranking from Figure 6 were used to identify isolates with the greatest relative virulence difference in the mouse and *G. mellonella* model.
